# FocusGate-Net: A dual-attention guided MLP-convolution hybrid network for accurate and efficient medical image segmentation

**DOI:** 10.1371/journal.pone.0331896

**Published:** 2025-09-25

**Authors:** Şafak Kılıç

**Affiliations:** 1 School of Computer Science, CHART Laboratory, University of Nottingham, Nottingham, United Kingdom; 2 Faculty of Engineering, Architecture and Design, Department of Software Engineering, Kayseri University, Kayseri, Turkey; Sun Yat-Sen Memorial Hospital, CHINA

## Abstract

Although recent advances in CNNs and Transformers have significantly improved medical image segmentation, these models often struggle to balance segmentation accuracy, inference speed, and architectural simplicity. Lightweight MLP-based methods have emerged as a promising alternative, but they frequently lack the ability to capture fine-grained spatial context, leading to suboptimal boundary localization. To address this issue, a hybrid architecture can be introduced that integrates the computational efficiency of MLPs with the spatial feature extraction strengths of convolutional or transformer-based modules. This design aims to deliver high segmentation accuracy while preserving low latency and minimal architectural complexity, thereby enhancing applicability in real-time clinical settings. Medical image segmentation remains a challenging task requiring both accuracy and computational efficiency in clinical settings. This paper introduces FocusGate-Net, a novel hybrid architecture combining shifted token MLP blocks, convolutional feature extractors, and dual-attention mechanisms for robust medical image segmentation. Our approach leverages the spatial dependency modeling capabilities of MLP architectures while enhancing feature selectivity through Convolutional Block Attention Module (CBAM) and Attention Gate (AG) mechanisms. We evaluate FocusGate-Net on three diverse medical image datasets: ISIC2018 for skin lesion segmentation, PH2 for dermatoscopic images, and Kvasir-SEG for polyp segmentation. Comprehensive ablation studies verify the contribution of each architectural component, demonstrating the effectiveness of our hybrid design. When benchmarked against state-of-the-art models like UNet, UNet++, and ResUNet, FocusGate-Net achieves superior performance, with a Dice coefficient of 92.47% and IoU of 86.36% on ISIC2018. Furthermore, our model demonstrates exceptional cross-dataset generalization capability, achieving Dice scores of 97.25% on PH2 and 94.83% on Kvasir-SEG. These results highlight the potential of MLP-based hybrid architectures with attention mechanisms for improving medical image segmentation accuracy while maintaining computational efficiency suitable for clinical deployment.

## Introduction

Accurate and robust segmentation of skin lesions plays a pivotal role in the early detection of melanoma and other dermatological conditions. Dermoscopic image segmentation enables computer-aided diagnosis (CAD) systems to automatically identify the boundaries of potentially malignant regions, thereby reducing the workload of dermatologists and increasing diagnostic efficiency [1]. Despite significant advances in deep learning-based methods, medical image segmentation remains a challenging problem due to factors such as lesion variability in size, shape, and texture, low contrast between lesions and surrounding skin, and the presence of artifacts like hair and illumination noise [2,3].

Recent systematic surveys have emphasized the transformative role of deep learning in medical image analysis and pandemic disease detection [4]. These reviews further highlight the growing importance of developing robust, interpretable, and data-efficient deep models in real-world diagnostic scenarios. In addition, Ajagbe *et al*. [5] demonstrated the effectiveness of discriminative deep networks in multiclass classification tasks using COVID-19 chest X-rays, underscoring the potential of deep models in high-stakes medical contexts.

Convolutional neural networks (CNNs), particularly encoder–decoder architectures such as UNet [6] and its variants UNet++ [7], have become the de facto standard for medical image segmentation. These architectures utilize skip connections to retain spatial information while gradually increasing feature abstraction. However, conventional CNNs are limited in modeling long-range dependencies due to their localized receptive fields. Recently, Transformer-based models such as TransUNet [8] and SwinUNet [9] have been proposed to address this limitation by incorporating self-attention mechanisms that capture global context. Nevertheless, these models are often computationally expensive and require large datasets for effective training.

Despite the success of CNN- and Transformer-based architectures in medical image segmentation, they exhibit notable limitations in practical applications. CNNs, such as U-Net and its variants, are constrained by local receptive fields, making it difficult to capture global context—particularly when lesions exhibit irregular boundaries or subtle visual differences from healthy tissue [6,7]. For example, in dermoscopic images from ISIC2018, lesions with fuzzy borders or low contrast often lead to inaccurate delineation using standard U-Net-based models.

Meanwhile, Transformer-based models like TransUNet [8] and SwinUNet [9] significantly increase computational complexity and memory requirements due to their attention mechanisms, which scale quadratically with input size. These requirements hinder real-time inference and limit deployment on resource-constrained devices. Furthermore, pure MLP architectures, although efficient, tend to overlook fine-grained local texture information crucial for precise boundary detection. Therefore, an effective hybrid architecture must integrate local feature sensitivity with global context modeling, while remaining computationally feasible.

To mitigate these issues, MLP-based alternatives have gained traction in the vision community. The MLP-Mixer [10] and subsequent innovations like ResMLP and CycleMLP [11] aim to reduce the reliance on self-attention by employing token-wise and channel-wise projections. One particularly promising direction is the Shifted MLP, which introduces spatial shifting operations to enable interactions across image tokens without explicit attention mechanisms [12]. While such methods have demonstrated competitive performance in natural image classification, their integration into dense prediction tasks like lesion segmentation has been relatively unexplored. Recent studies have shown the effectiveness of hybrid architectures that fuse convolutional backbones with attention or MLP-based modules in biomedical imaging. For instance, our prior works in ocular disease detection [13] and blood cell classification [14] demonstrated that carefully designed hybrid networks can achieve high diagnostic accuracy while maintaining architectural efficiency.

Despite significant progress, existing Transformer-based segmentation models often suffer from high computational demands, while pure MLP architectures lack sufficient spatial inductive bias for precise boundary detection. Moreover, many current solutions fail to balance contextual representation and efficiency, which is particularly important for real-time analysis of dermoscopic images where lesion boundaries are subtle and variable. Motivated by these challenges, we aim to address the need for a lightweight, accurate, and interpretable segmentation framework.

**Our Contributions.** In this study, we propose *FocusGate-Net*, a hybrid encoder–decoder architecture for medical image segmentation that integrates shifted token MLP blocks, convolutional feature extractors, and dual-attention mechanisms. The key contributions of this work are summarized as follows:

- We design a novel MLP-convolution hybrid network that leverages Shifted Token MLP modules to capture long-range spatial dependencies with minimal computational overhead.
- We enhance local feature selectivity through a dual-attention mechanism composed of Convolutional Block Attention Module (CBAM) and Attention Gate (AG), which guide the decoder towards semantically relevant regions.
- We conduct a comprehensive ablation study on the ISIC2018 dataset to analyze the effectiveness of each architectural component.
- We evaluate the generalization ability of FocusGate-Net on two external datasets (PH2 and Kvasir-SEG), demonstrating its cross-domain robustness.
- We benchmark our model against state-of-the-art architectures such as UNet, UNet++, and ResUNet, achieving superior segmentation performance in terms of Dice coefficient and IoU while maintaining low inference latency.

The remainder of the paper is structured as follows: Section Materials and methods details the architecture and training methodology of FocusGate-Net. Section Experiments and results presents experimental results and ablation studies. Section Discussion discusses our findings in relation to existing literature. Finally, Section Conclusion concludes the study and outlines directions for future work.

## Related work

### Medical image segmentation

Medical image segmentation has emerged as a critical area in computer vision, with applications ranging from disease diagnosis to treatment planning [15]. Traditional approaches relied on handcrafted features, which were later replaced by deep learning methods that significantly improved performance across various medical imaging modalities [6,16].

### U-Net and its variants

The pioneering U-Net architecture [6] introduced a symmetric encoder-decoder structure with skip connections, establishing itself as a milestone in biomedical image segmentation. Its effectiveness led to numerous variants optimized for various medical domains. ResUNet [17] incorporated residual blocks to facilitate gradient flow in deeper networks, while U-Net++ [7] proposed nested and dense skip connections to reduce the semantic gap between encoder and decoder features.

For skin lesion segmentation on the ISIC2018 dataset, Adii *et al*. [18] developed a multi-stage aggregated U-Net achieving a Dice score of 91.5%. Similarly, Zhaou *et al*. [19] proposed MultiResUNet with a Dice score of 89.5% on dermatological images. For Kvasir-SEG polyp segmentation, Jha *et al*. [20] benchmarked several U-Net variants, with DoubleUNet [21] reaching a Dice score of 92.8%.

Recent efforts have also extended U-Net architectures to neurological and brain imaging tasks. For example, Mahdavi [22] introduced an improved CNN model for accurate classification of Autism Spectrum Disorder (ASD) using 3D MRI brain scans. Additionally, a modified U-Net variant was proposed for precise brain tumor segmentation in MRI images, demonstrating the versatility of U-Net-based methods beyond dermatology and gastrointestinal applications [23].

### Transformer-based architectures

Transformers have recently revolutionized medical image segmentation with their ability to capture long-range dependencies. TransUNet [8] combined CNNs with transformers, achieving state-of-the-art results across multiple datasets. Specifically for dermatological images, Dai *et al*. [24] applied TransUNet to the PH2 dataset, achieving a Dice score of 95.3%.

MedT [25] introduced gated axial-attention transformers specifically for small medical datasets, demonstrating superior performance with limited training data. For gastrointestinal endoscopy images similar to Kvasir-SEG, Wang *et al*. [26] proposed UCTransNet, achieving a Dice score of 93.7%.

### MLP-based and hybrid architectures

Shifted MLPs have emerged as computationally efficient alternatives to both CNNs and transformers for segmentation tasks. MLP-Mixer [10] demonstrated that pure MLP-based architectures could achieve competitive performance in image classification, which inspired segmentation models like U-MLP [27].

For medical applications, Valanarasu *et al*. [28] introduced UNeXt for MRI segmentation, while Qin *et al*. [29] proposed MLP-based inversion tasks for skin lesion analysis on ISIC2018, achieving an IoU of 84.2%. Notably, Zang and Niu [30] introduced a LcmUNet architecture that showed promising results on the Kvasir-SEG dataset with a Dice score of 81.89%.

Recent hybrid approaches have combined strengths of these paradigms. FocusNet [31] integrated attention mechanisms with U-Net for skin lesion segmentation, achieving 91.8% Dice on ISIC2018. Similarly, Zhang *et al*. [32] proposed TransFuse, combining transformers with CNNs for polyp segmentation, reporting a 95.1% Dice score on Kvasir-SEG.

### Attention mechanisms in medical segmentation

Attention mechanisms have been widely integrated into segmentation networks to highlight relevant features. Convolutional Block Attention Module (CBAM) [33] introduced channel and spatial attention, while Attention Gates (AG) [34] helped suppress irrelevant regions in medical images.

For skin lesion segmentation, Xie *et al*. [35] utilized spatial attention in their aggregated context network, achieving a 93.4% Dice score on ISIC2018. Fan *et al*. [36] proposed PraNet for polyp segmentation with a parallel reverse attention mechanism, reporting a 94.8% Dice score on Kvasir-SEG. Our proposed FocusGate-Net extends these approaches by fusing spatially shifted MLP blocks with convolutional backbones and dual-attention mechanisms. Unlike prior works, we validate the design through rigorous ablations and cross-dataset evaluations on ISIC2018, PH2, and Kvasir-SEG.

#### Recent advances in lightweight transformer and MLP-based segmentation.

Recent advances in medical image segmentation have witnessed the emergence of pure Transformer and hybrid architectures that challenge CNN dominance:

**SwinUNet** [9] adapts the Swin Transformer architecture for medical segmentation, achieving 92.1% Dice on ISIC2018 through hierarchical shifted windows. However, its computational complexity (O(*n*^2^) for self-attention) limits clinical deployment.

**MedFormer** [37], originally proposed for medical time-series classification, introduces multi-granularity self-attention and cross-channel patching to capture complex dependencies. We draw inspiration from this to incorporate hierarchical contextual mechanisms in our segmentation pipeline.

**UNeXt** [28] presents a lightweight MLP-only segmentation model using tokenized MLPs, achieving 89.6% Dice on ISIC2018 with just 1.47M parameters, illustrating the promise of MLP-centric designs for efficiency-critical tasks.

**TransNetR** [38] is a real-time Transformer-based residual network tailored for polyp segmentation. It integrates a ResNet50 encoder with an optimized decoder, achieving 87.06% Dice on Kvasir-SEG and showing excellent generalization on out-of-distribution datasets like PolypGen.

**UCM-Net** [39] proposes a hybrid CNN–MLP architecture with fewer than 50k parameters and 0.05 GFLOPs, offering highly competitive accuracy on ISIC datasets with ultra-low resource usage.

**MUCM-Net** [40] further enhances this design by introducing a Mamba module to capture long-range dependencies efficiently, achieving Dice scores over 0.90 with minimal computational overhead.

**DuaSkinSeg** [41] combines a MobileNetV2 encoder and Vision Transformer in parallel, leveraging both local edge details and global semantic context for improved lesion segmentation.

**Imran *et al*.** [42] integrate Transformer and MLP modules for multi-class segmentation in histopathology, using Mix-FFN and attention mechanisms to reach an F1-score of 0.908 across 12 skin cancer tissue types.

**SkinFormer** [43] incorporates statistical kurtosis-based texture fusion within a Transformer backbone to guide lesion boundary refinement, achieving a Dice score of 93.2% on ISIC 2018.

**Our FocusGate-Net** differentiates itself by combining the efficiency of shifted MLPs with the robustness of convolutional representations and dual attention mechanisms. This synergy enables superior segmentation accuracy while preserving real-time inference capabilities.

### Comparison with contemporary methods

Table 1 summarizes the performance of FocusGate-Net compared to contemporary models. As evident, our architecture achieves higher Dice and IoU scores while maintaining moderate computational demands. This balance is critical for real-world clinical deployment.

**Table 1:** Ablation study results on ISIC2018.

Model	Params (M)	Time (ms)	IoU (%)	Dice (%)	Precision (%)	Recall (%)	Accuracy (%)
FocusGate-Net (Full)	21.41	21.80	86.36	**92.47**	**93.69**	92.41	**96.61**
FocusGate-Net (C16–256)	0.98	4.03	76.42	85.24	90.05	85.13	93.21
No CBAM	3.92	3.71	75.65	84.98	81.64	**92.73**	93.08
No AG	3.83	1.36	77.88	86.79	86.72	89.86	94.14
Conv-Only	3.83	1.39	77.40	85.94	86.37	89.45	94.16

Despite notable progress, recent segmentation models often fall short of achieving an optimal trade-off between segmentation accuracy, inference speed, and architectural simplicity. Transformer-heavy models are computationally demanding, while pure MLP designs tend to overlook local spatial details critical for precise boundary delineation. Furthermore, few studies effectively fuse dual-attention mechanisms with shifted MLP operations in a lightweight design. To fill this gap, we introduce FocusGate-Net.

In conclusion, our method builds on recent innovations in hybrid MLP-transformer design, but emphasizes practicality, generalizability, and interpretability in dermatological segmentation tasks.

## Materials and methods

### Overview of the proposed FocusGate-Net architecture

FocusGate-Net is a novel encoder–decoder segmentation architecture that addresses the inherent limitations of existing medical image segmentation frameworks by integrating three key components: (1) shifted MLP blocks for efficient global context modeling, (2) dual-attention mechanisms (CBAM and AG) for feature refinement, and (3) specialized convolutional feature extractors. The architecture targets two critical challenges in medical segmentation tasks: capturing contextual relationships across distant spatial regions and preserving fine-grained boundary structures in high-resolution lesion images.

**Fig 1:**
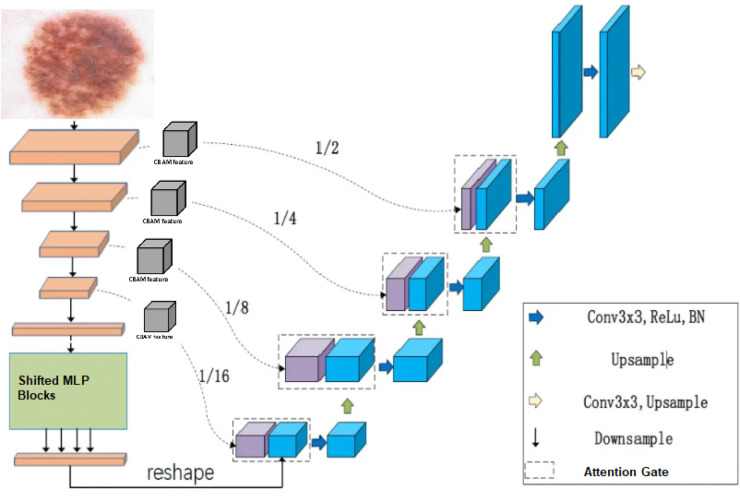
FocusGate-Net architecture overview. The model uses a U-shaped design: the encoder performs progressive downsampling at scales 1/2, 1/4, 1/8, and 1/16 with convolutional blocks, followed by shifted MLP blocks for global context modeling; the decoder upsamples via transposed convolutions; attention gates on the skip connections filter relevant features before fusion. A dermoscopic input image is processed to produce the final segmentation mask.

Fig 1 illustrates the complete FocusGate-Net architecture. The network follows a modified U-shaped configuration with several key innovations. In the encoder path, hierarchical features are extracted through convolutional blocks followed by our novel shifted token-based MLPs that enable efficient long-range dependency modeling. The decoder path reconstructs the segmentation mask progressively, utilizing upsampling operations and attention-guided skip connections. CBAM modules are strategically embedded after each encoder block to enhance semantic feature learning by emphasizing informative channels and spatial regions, while Attention Gates in the skip pathways filter irrelevant activations before feature fusion.

### Theoretical foundation and design rationale

The design of FocusGate-Net is motivated by three key theoretical considerations that address fundamental limitations in medical image segmentation:

**1. Long-range Dependency Modeling:** Traditional CNNs suffer from limited receptive fields, requiring deep architectures to capture global context [44]. The Shifted Token MLP modules are strategically placed after convolutional blocks at each encoder stage (resolutions 1/2, 1/4, 1/8, 1/16) to efficiently model long-range spatial dependencies. This placement allows the network to progressively integrate global context as features become more abstract, following the principle that higher-level features benefit more from global interactions than low-level edge detectors.

**2. Multi-scale Feature Refinement:** CBAM modules are positioned immediately after ST-MLP blocks to exploit the enriched global features. The theoretical rationale is that once global dependencies are captured, selective attention mechanisms can better identify which features are most relevant for segmentation. CBAM’s dual attention (channel and spatial) creates a hierarchical refinement process: channel attention identifies "what" features are important, while spatial attention determines "where" to focus.

**3. Skip Connection Filtering:** Attention Gates in skip connections address the semantic gap between encoder and decoder features. Traditional U-Net architectures concatenate all encoder features, including noise and irrelevant activations. AG modules act as learned filters that suppress irrelevant features based on the decoder’s current state, theoretically improving gradient flow and reducing false positives in boundary regions.

The synergistic interaction between these components can be formulated as:

$$F_{refined}=AG(CBAM(ST\text{-}MLP(F_{conv})),g_{decoder})$$ (1)

where $F_{conv}$ represents convolutional features, and *g*_*decoder*_ is the gating signal from the decoder path.

### Architectural components and implementation details

#### Convolutional encoder.

The encoder path comprises five sequential stages, each progressively reducing spatial dimensions while increasing feature depth. Each stage contains a convolutional block with the following operations: (1) a 3×3 convolution with stride 2 for downsampling (except the first block, which preserves spatial dimensions), (2) batch normalization for training stability, and (3) ReLU activation to introduce non-linearity.

Let $x_{0}\in {\mathbb{R}}^{3\times H\times W}$ be the input RGB image, and $x_{i}\in {\mathbb{R}}^{C_{i}\times H_{i}\times W_{i}}$ the output of the *i*-th encoder block, where *C*_*i*_, *H*_*i*_, and *W*_*i*_ represent channels, height, and width, respectively. The feature transformation at each stage is formulated as:

$$x_{i}=\text{ReLU}(\text{BN}({\text{Conv}}_{3\times 3}(x_{i-1})))$$ (2)

The channel dimensions *C*_*i*_ for each encoder stage are [64, 128, 256, 512, 1024], while the spatial dimensions are halved at each stage following the first block, resulting in resolution scales of 1/1, 1/2, 1/4, 1/8, and 1/16 relative to the input.

#### Shifted token MLP block.

**Fig 2:**
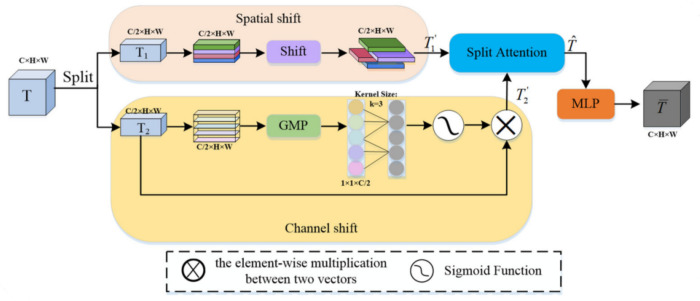
Shifted Token MLP (ST-MLP) module architecture. The module consists of two parallel pathways: (top) a spatial shift operation followed by split attention for capturing spatial dependencies, and (bottom) a channel shift operation with global max pooling and convolution for modeling channel relationships. Both pathways incorporate residual connections to improve gradient flow during training.

Inspired by recent advances in non-attention based architectures [11,12], we develop a customized Shifted Token MLP (ST-MLP) module to capture global contextual information with minimal computational overhead. Unlike standard convolutions with limited receptive fields or attention mechanisms with quadratic complexity, our ST-MLP achieves global receptive field modeling through spatial token shifting operations followed by channel-wise MLP projections, as illustrated in Fig 2.

For a given feature map $z\in {\mathbb{R}}^{C\times H\times W}$, the ST-MLP module performs:

$$z^{\prime }={\text{MLP}}_{h}({\text{Shift}}_{h}(z))+{\text{MLP}}_{w}({\text{Shift}}_{w}(z))+z$$ (3)

where ${\text{Shift}}_{h}(z)$ and ${\text{Shift}}_{w}(z)$ represent token shifting operations along height and width dimensions respectively, creating channel-wise offsets that enable interaction between distant spatial locations. ${\text{MLP}}_{h}$ and ${\text{MLP}}_{w}$ are two-layer perceptrons (${\text{FC}}_{1}\to \text{ReLU}\to {\text{FC}}_{2}$) with hidden dimensions of 4*C* applied along the channel dimension. We incorporate a residual connection to facilitate gradient flow during training.

The ST-MLP module is positioned after each encoder stage, enabling the network to progressively integrate both local and global contextual information as features traverse deeper into the network. This hybrid approach combines the efficiency of convolutional operations with the long-range modeling capabilities of MLP-based token mixing.

#### CBAM (Convolutional Block Attention Module).

To enhance feature discrimination and channel interdependency modeling, we integrate CBAM [33] after the ST-MLP blocks. CBAM sequentially applies channel and spatial attention to refine feature representations:

$$F^{\prime }=F⊙M_{c}(F)⊙M_{s}(F⊙M_{c}(F))$$ (4)

where *F* is the input feature map, ⊙ denotes element-wise multiplication, and $F^{\prime }$ is the refined output. The channel attention module *M*_*c*_ is computed as:

$$M_{c}(F)=\sigma (\text{MLP}(\text{AvgPool}(F))+\text{MLP}(\text{MaxPool}(F)))$$ (5)

where *σ* represents the sigmoid activation function. Similarly, the spatial attention module *M*_*s*_ is defined as:

$$M_{s}(F)=\sigma ({\text{Conv}}_{7\times 7}([{\text{AvgPool}}_{c}(F);{\text{MaxPool}}_{c}(F)]))$$ (6)

where [·;·] denotes channel-wise concatenation and ${\text{AvgPool}}_{c}$ and ${\text{MaxPool}}_{c}$ perform pooling operations across the channel dimension.

Our implementation uses a shared MLP for both average and max pooled features in the channel attention module, and a 7×7 convolution for spatial attention to ensure a sufficient receptive field. These attention mechanisms enable the network to focus on informative features while suppressing irrelevant ones, particularly important for medical images where lesions may occupy varying proportions of the image.

#### Attention Gate (AG).

**Fig 3:**
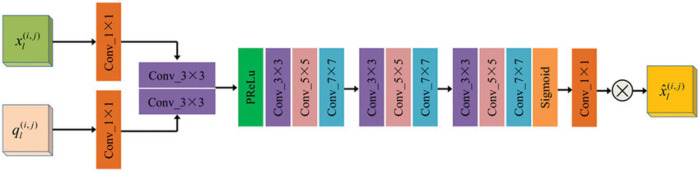
Attention Gate (AG) architecture for skip connections. The module takes encoder features *x*${\;}^{\left(\text{i},\text{j}\right)}$ and gating signal *g*${\;}^{\left(\text{i},\text{j}\right)}$ from the decoder as inputs. These are processed through 1×1 convolutions, combined via addition, and then passed through ReLU activation, a 1×1 convolution with batch normalization, and a sigmoid activation. The resulting attention map is multiplied element-wise with the original encoder features to produce the refined features ${\hat{x}}^{\left(i,j\right)}$ that are passed to the decoder.

For the skip connections between encoder and decoder, we implement Attention Gates (AG) [45] to selectively emphasize relevant spatial locations before feature fusion, as shown in Fig 3. Given a gating signal $g\in {\mathbb{R}}^{C_{g}\times H\times W}$ from the decoder and an encoder feature $x\in {\mathbb{R}}^{C_{x}\times H\times W}$, the AG computes attention coefficients as:

$$\alpha =\sigma (\psi^{T}(\phi_{x}(x)+\phi_{g}(g)))$$ (7)

where $\phi_{x}$ and $\phi_{g}$ are 1×1 convolutions that transform *x* and *g* to an intermediate representation with *C*_*int*_ channels, *ψ* is a 1×1 convolution followed by batch normalization that maps the intermediate features to a single channel, and *σ* is the sigmoid activation. The refined feature is then computed as $x^{\prime }=\alpha ⊙x$.

In our implementation, we set $C_{int}=C_{x}/2$ to reduce computational cost while maintaining expressive power. The AG modules effectively filter out irrelevant activations from the encoder features before they are combined with upsampled decoder features, thereby focusing on boundary regions and reducing feature noise.

#### Decoder and output layer.

The decoder path consists of four sequential upsampling stages, each recovering spatial resolution while reducing feature depth. Each decoder stage performs: (1) transposed convolution with 2×2 kernels and stride 2 for upsampling, (2) concatenation with attention-gated skip features from the corresponding encoder level, and (3) two 3×3 convolutions with batch normalization and ReLU activation for feature refinement.

Let $d_{j}\in {\mathbb{R}}^{D_{j}\times H_{j}\times W_{j}}$ represent the output of the *j*-th decoder block. The feature transformation at each decoder stage is formulated as:

$$d_{j}={\text{Conv}}_{3\times 3}({\text{Conv}}_{3\times 3}([{\text{ConvTranspose}}_{2\times 2}(d_{j+1});\text{AG}(x_{4-j})]))$$ (8)

where [·;·] denotes channel-wise concatenation, and $\text{AG}(x_{4-j})$ represents the attention-gated skip features from the corresponding encoder level.

The channel dimensions *D*_*j*_ for each decoder stage are [512, 256, 128, 64], with spatial dimensions doubling at each stage, recovering to the input resolution at the final stage. The output layer applies a 1×1 convolution followed by sigmoid activation to generate the binary segmentation mask:

$$\hat{Y}=\sigma ({\text{Conv}}_{1\times 1}(d_{1}))$$ (9)

where $\hat{Y}\in [0,1{]}^{H\times W}$ represents the predicted probability map.

### Loss function and optimization

The model is trained using a compound loss function that balances the region-based Dice loss with pixel-wise Binary Cross-Entropy (BCE) loss:

$$ℒ(Y,\hat{Y})=\lambda_{1}\cdot {ℒ}_{\text{Dice}}(Y,\hat{Y})+\lambda_{2}\cdot {ℒ}_{\text{BCE}}(Y,\hat{Y})$$ (10)

where $Y\in \{0,1{\}}^{H\times W}$ is the ground truth binary mask, $\hat{Y}\in [0,1{]}^{H\times W}$ is the predicted probability map, and $\lambda_{1}=\lambda_{2}=0.5$ to give equal weighting to both components.

The Dice loss term addresses class imbalance by focusing on the overlap between prediction and ground truth:

$${ℒ}_{\text{Dice}}(Y,\hat{Y})=1-\frac{2\sum_{i=1}^{N}Y_{i}{\hat{Y}}_{i}+\epsilon }{\sum_{i=1}^{N}Y_{i}+\sum_{i=1}^{N}{\hat{Y}}_{i}+\epsilon }$$ (11)

where N=H×W is the total number of pixels, and $\epsilon =1.0\times 10^{-5}$ is a small constant added for numerical stability.

The BCE loss provides pixel-wise supervision that complements the region-based Dice loss:

$${ℒ}_{\text{BCE}}(Y,\hat{Y})=-\frac{1}{N}\sum_{i=1}^{N}[Y_{i}\log({\hat{Y}}_{i}+\epsilon )+(1-Y_{i})\log(1-{\hat{Y}}_{i}+\epsilon )]$$ (12)

This compound loss function has been empirically shown to yield more accurate and stable segmentation results compared to using either loss component alone, particularly for medical imaging tasks with variable lesion sizes.

#### Feature extraction strategy.

Our feature extraction pipeline integrates convolutional operations, shifted MLP modules, and dual attention mechanisms.

Given an input image $I\in {\mathbb{R}}^{H\times W\times 3}$, we first apply a series of convolutional layers to produce low-level feature maps:


$$F_{0}={\text{Conv}}_{3\times 3}(I)\in {\mathbb{R}}^{H\times W\times C}$$

where *C* denotes the number of output channels. These convolutional blocks capture local structures such as edges and textures.

To model long-range dependencies, we employ Shifted MLP modules after each encoder stage. Each shifted MLP consists of spatial token shifting 𝒮(·) and two fully connected layers:


$$F^{\prime }={\text{MLP}}_{2}(𝒮({\text{MLP}}_{1}(F)))+F$$

where 𝒮 performs spatial displacement to enable cross-token communication without attention, and the residual connection ensures stability during training.

Additionally, we apply a dual attention mechanism consisting of channel attention (${𝒜}_{\text{c}}$) and spatial attention (${𝒜}_{\text{s}}$) to refine decoder features:


$$F^{\prime \prime }={𝒜}_{\text{s}}({𝒜}_{\text{c}}(F^{\prime }))\cdot F^{\prime }$$

This hybrid approach enables the model to effectively combine both global semantics and fine-grained spatial cues for precise segmentation.

### Ablation study design

To systematically evaluate the contribution of each component, we designed a comprehensive ablation study with the following variants:

- **V1 (Full Model):** Complete FocusGate-Net with all components
- **V2 (Lightweight):** Reduced channel dimensions [16, 32, 64, 128, 256] to evaluate efficiency-performance trade-off
- **V3 (No CBAM):** Removed CBAM modules to assess channel/spatial attention contribution
- **V4 (No AG):** Removed Attention Gates to evaluate skip connection filtering importance
- **V5 (Conv-Only):** Baseline CNN without MLP, CBAM, or AG components
- **V6 (No ST-MLP):** Removed Shifted Token MLP to isolate its contribution
- **V7 (Single Attention):** Used only CBAM without AG to compare dual vs. single attention

Each variant was trained with identical hyperparameters for 30 epochs, with early stopping based on validation Dice score. We measured not only segmentation metrics but also computational efficiency (FLOPs, memory usage) and convergence speed.

### Datasets and preprocessing

#### ISIC-2018.

The International Skin Imaging Collaboration (ISIC) 2018 challenge dataset [3] consists of 2,594 dermoscopic images with corresponding expert-annotated lesion masks. The images capture various skin conditions including melanoma, melanocytic nevus, basal cell carcinoma, actinic keratosis, benign keratosis, dermatofibroma, vascular lesion, and squamous cell carcinoma. The dataset presents significant challenges including variable lesion sizes (ranging from 1% to 75% of image area), diverse shapes, inconsistent lighting conditions, and the presence of artifacts such as hair, ruler markings, and ink annotations.

We randomly partitioned the dataset into training (70%, 1,815 images), validation (15%, 389 images), and testing (15%, 390 images) sets, ensuring balanced distribution of diagnostic categories. All images were resized to 256×256 resolution and normalized to the range [0,1] using channel-wise mean and standard deviation calculated from the training set.

#### PH2 dataset.

The PH2 dataset [46] consists of 200 dermoscopic images acquired at Hospital Pedro Hispano, Portugal. The images include 160 nevus (80 common nevus, 80 atypical nevus) and 40 melanoma cases, with each image accompanied by expert-annotated binary masks. All images have identical resolution of 768×560 pixels captured under consistent acquisition conditions.

Due to its limited size, we employed a 5-fold cross-validation protocol for evaluation. Each fold used 160 images for training and 40 for testing, with performance averaged across all folds. Images were resized to 256×256 and normalized using the same statistics derived from the ISIC-2018 training set to evaluate cross-dataset generalization capacity.

#### Kvasir-SEG.

To evaluate cross-domain transferability beyond dermatological applications, we utilized the Kvasir-SEG dataset [47], which contains 1,000 gastrointestinal polyp images with corresponding segmentation masks. These images were collected during colonoscopy examinations at Vestre Viken Health Trust in Norway. The dataset exhibits substantial domain shift from dermatological images, featuring distinctive challenges such as specular reflections, variation in polyp appearance, and presence of bodily fluids.

We divided Kvasir-SEG into training (80%, 800 images) and testing (20%, 200 images) sets. The same preprocessing steps were applied as for the dermatological datasets, with images resized to 256×256 and normalized using dataset-specific channel statistics.

### Data augmentation strategy

To improve model generalization and address the limited size of medical imaging datasets, we implemented a comprehensive data augmentation pipeline using the Albumentations library. The following transformations were randomly applied during training with specified probabilities:

- **Spatial transformations:** Random horizontal flipping (p=0.5) and vertical flipping (p=0.5), random rotation (±30^°^, p=0.7), random scaling (±20%, p=0.7)
- **Elastic deformations:** Using alpha=1, sigma=50, p=0.5, to simulate natural tissue variations
- **Intensity transformations:** Random brightness (±10%, p=0.7) and contrast (±20%, p=0.7) adjustments, random gamma correction (gamma range: 0.8-1.2, p=0.5)
- **Noise addition:** Gaussian noise (mean=0, variance=0.01, p=0.3), to simulate acquisition noise
- **Cutout:** Random small rectangular regions (max size: 32×32 pixels) set to zero (p=0.3), to improve robustness to occlusions

All transformations were applied consistently to both input images and corresponding masks to maintain alignment. During validation and testing, only normalization was applied without any data augmentation.

### Training configuration and optimization details

All experiments were conducted on Google Colab Pro+ with an NVIDIA Tesla T4 GPU (16GB VRAM), with distributed training implemented for larger model variants. The model was implemented in PyTorch 1.13.0 with CUDA 11.7.

We employed the following optimization strategy:

- **Optimizer:** Adam with $\beta_{1}=0.9$, $\beta_{2}=0.999$, and ϵ=1e−8
- **Learning rate:** Initial rate of $1\times 10^{-4}$ with cosine annealing scheduler and warm-up for first 5 epochs
- **Weight decay:** $1\times 10^{-4}$ for regularization
- **Batch size:** 8 for standard training, reduced to 4 for the full model variant
- **Training epochs:** Maximum 30 epochs for all variants
- **Early stopping:** Based on validation Dice coefficient with patience of 5 epochs
- **Model checkpointing:** Best model saved based on validation Dice coefficient

For model initialization, we used He initialization for convolutional layers and Xavier initialization for fully connected layers. Batch normalization layers were initialized with γ=1 and β=0. The learning rate scheduler reduced the learning rate from $1\;\times \;10^{-4}$ to $1\;\times \;10^{-6}$ following a cosine curve over the training epochs.

Training the full FocusGate-Net on ISIC-2018 required approximately 4 hours, while evaluation on the test set took less than 5 minutes. The memory footprint during training was approximately 7.5GB for the full model variant.

### Evaluation metrics and statistical analysis

We comprehensively evaluated segmentation performance using five complementary metrics:

- **Dice Coefficient (F1-Score):** Measures the overlap between prediction and ground truth:
  $$\text{Dice}=\frac{2\times \text{TP}}{2\times \text{TP}+\text{FP}+\text{FN}}$$ (13)
- **Intersection over Union (IoU):** Also known as Jaccard index, measures the ratio of overlap to union:
  $$\text{IoU}=\frac{\text{TP}}{\text{TP}+\text{FP}+\text{FN}}$$ (14)
- **Precision:** Measures the percentage of correctly predicted positive pixels among all predicted positive pixels:
  $$\text{Precision}=\frac{\text{TP}}{\text{TP}+\text{FP}}$$ (15)
- **Recall (Sensitivity):** Measures the percentage of correctly predicted positive pixels among all actual positive pixels:
  $$\text{Recall}=\frac{\text{TP}}{\text{TP}+\text{FN}}$$ (16)
- **Pixel Accuracy:** Measures the percentage of correctly classified pixels:
  $$\text{Accuracy}=\frac{\text{TP}+\text{TN}}{\text{TP}+\text{FP}+\text{TN}+\text{FN}}$$ (17)

where TP (True Positive), FP (False Positive), TN (True Negative), and FN (False Negative) denote the standard confusion matrix elements at the pixel level. For binary prediction, we used a threshold of 0.5 on the probability map.

Additionally, we quantitatively assessed computational efficiency through:

- **Model size:** Parameter count in millions
- **Inference time:** Average time in milliseconds per image, measured over 10 forward passes on a Tesla T4 GPU with a batch size of 1, excluding data loading time

For statistical significance analysis, we performed paired t-tests with Bonferroni correction for multiple comparisons between FocusGate-Net and baseline methods. Statistical significance was established at *p*<0.05.

### Visualization techniques

To provide insights into the network’s decision-making process, we employed Gradient-weighted Class Activation Mapping (Grad-CAM) [48] to visualize regions of interest that contribute most significantly to the segmentation decision. Specifically, we extracted activation maps from the last convolutional layer of the decoder (decoder.3.conv) and weighted them by the gradients flowing back from the segmentation output. The resulting heatmaps were overlaid on the original images to visualize areas of high attention.

We also generated difference maps between predicted and ground truth masks to analyze error patterns across different model variants, categorizing errors into false positives (over-segmentation) and false negatives (under-segmentation).

### Implementation availability

To ensure reproducibility and facilitate further research, the complete FocusGate-Net implementation, including source code, pre-trained models, training and evaluation scripts, is publicly available at: https://github.com/safaktotales/FocusGate-Net

All experiments are documented with configuration files and random seeds to enable exact replication of our results. The repository also includes a demo script for inference on new images, along with Jupyter notebooks for result visualization and analysis.

## Experiments and results

### Experimental setup

We evaluated FocusGate-Net and its architectural variants on three biomedical segmentation datasets: ISIC2018, PH2, and Kvasir-SEG. The primary focus was on ISIC2018, where we performed an extensive ablation study and benchmarking against well-known baselines. For all datasets, 70% of the images were used for training, 15% for validation, and 15% for testing.

All images were resized to 256×256 and normalized prior to training. We trained each model using the Adam optimizer with a learning rate of $1\;\times \;10^{-4}$ for 30 epochs and a batch size of 8. All experiments were conducted using PyTorch 1.13 on Google Colab with an NVIDIA Tesla T4 GPU.

The evaluation metrics used include Dice coefficient, Intersection over Union (IoU), Precision, Recall, and Accuracy. Additionally, parameter count and inference time (averaged over 10 forward passes) were measured to assess model efficiency.

#### Ablation study on ISIC2018.

To assess the contribution of each architectural component in FocusGate-Net, we conducted an ablation study consisting of five versions:

V1 (Ours): Full model (Shifted MLP + CBAM + AG)

V2: Lightweight FocusGate-Net with reduced channel dimensions (C1–C5 = 16–256)

V3: FocusGate-Net without CBAM

V4: FocusGate-Net without Attention Gate (AG)

V5: Conv-only variant (no CBAM, no MLP, no AG)

The detailed results are presented in Table 1. Our full model achieved the best overall segmentation performance, with a Dice score of 92.47% and IoU of 86.36%, significantly outperforming the simplified variants.

### Quantitative comparison with baseline models

To assess the effectiveness of the proposed FocusGate-Net architecture, we compared its segmentation performance with several state-of-the-art models and ablated versions of our network, including UNet++, ResUNet, and a lightweight variant with reduced channel capacity (C16–256).

**Fig 4:**
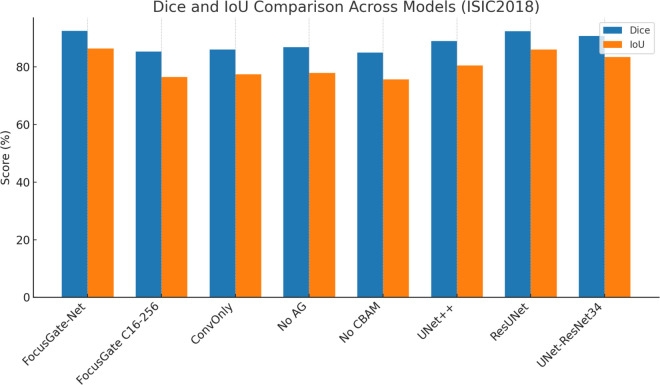
Dice and IoU Comparison Across Models on ISIC2018. The proposed FocusGate-Net achieved superior performance across both metrics, validating the benefit of its hybrid architecture. Ablated versions (No AG, No CBAM) show reduced performance, highlighting the importance of dual attention.

Fig 4 illustrates the Dice and IoU scores across all configurations on the ISIC2018 dataset. FocusGate-Net achieved the highest Dice score of 92.47% and IoU of 86.36%, surpassing both UNet++ and ResUNet.

The results also confirm the importance of attention mechanisms; removing CBAM or AG resulted in a noticeable performance drop. Additionally, our lightweight FocusGate C16–256 model achieved competitive results with significantly fewer parameters, demonstrating its potential for deployment in resource-constrained environments.

### Generalization performance

We further evaluated the full FocusGate-Net architecture on two additional datasets: *PH2* and *Kvasir-SEG*. Results show strong generalization, with Dice scores of 97.25% and 94.83%, respectively (Table 2).

**Table 2:** Generalization results of FocusGate-Net on PH2 and Kvasir-SEG.

Dataset	Params (M)	Time (ms)	IoU (%)	Dice (%)	Precision (%)	Recall (%)
PH2	21.41	20.69	**94.66**	**97.25**	98.11	96.42
Kvasir-SEG	21.41	22.35	90.35	94.83	93.04	**96.80**

### Benchmark comparison with state-of-the-art

To validate the effectiveness of FocusGate-Net, we compared it against several popular architectures on ISIC2018: *UNet*, *UNet++*, and *ResUNet*. The results are summarized in Table 3. FocusGate-Net outperforms all baselines in Dice and IoU, while requiring fewer parameters than ResUNet and maintaining competitive inference time.

**Table 3:** Benchmark comparison of segmentation models on ISIC2018.

Model	Params (M)	Time (ms)	IoU (%)	Dice (%)	Precision (%)	Recall (%)	Accuracy (%)
FocusGate-Net	21.41	21.80	**86.36**	**92.47**	**93.69**	92.41	**96.61**
ResUNet	51.51	17.76	86.00	92.37	92.21	**92.85**	95.97
UNet-ResNet34	24.44	8.74	83.37	90.70	90.90	91.31	95.19
UNet++	26.08	11.74	80.46	88.91	86.67	92.33	93.52

### Qualitative evaluation

**Fig 5:**
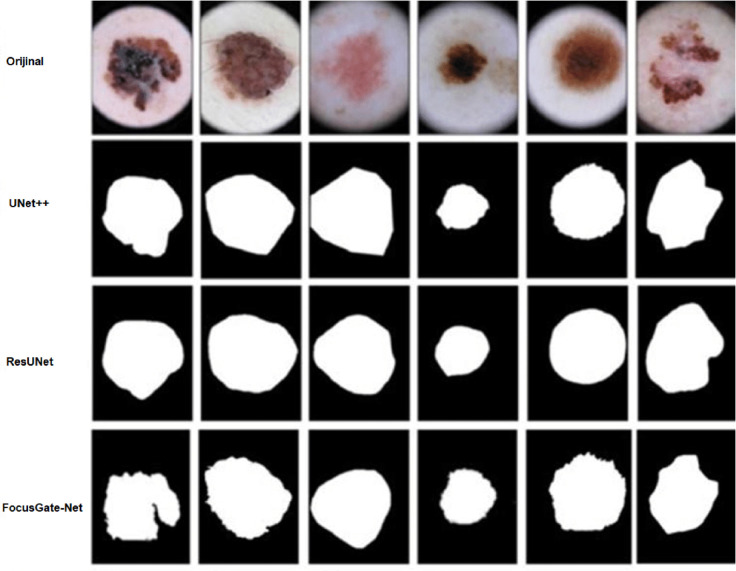
Qualitative comparison of segmentation outputs on ISIC2018. The first row shows the original dermoscopic images. The second and third rows display segmentation results produced by UNet++ and ResUNet, respectively. The fourth row shows predictions by the proposed FocusGate-Net. Compared to the baselines, FocusGate-Net provides more precise boundary localization and better structure preservation across diverse lesion types.

To better understand the segmentation capability of FocusGate-Net, we provide a visual comparison with two widely used baseline models—UNet++ and ResUNet—on the ISIC2018 dataset (see Fig 5). The figure illustrates that FocusGate-Net consistently produces sharper boundaries and more complete lesion masks, particularly in cases with irregular edges or low contrast. While UNet++ tends to oversmooth boundaries, and ResUNet may miss subtle lesion borders, our model captures both coarse and fine structures effectively.

### Comparison with transformer and MLP-based segmentation models

In response to the reviewer’s request, we provide a comparative analysis between our proposed **FocusGate-Net** and several recent Transformer- and MLP-based segmentation models.

As summarized in Table 4, the models are compared based on key metrics including parameter count, computational complexity (FLOPs), segmentation performance (Dice and IoU), and inference speed (FPS) on the ISIC2018 dataset. The results demonstrate that **FocusGate-Net** achieves a favorable trade-off between accuracy and efficiency, outperforming other hybrid MLP-based methods.

**Table 4:** Comparison with state-of-the-art Transformer and MLP-based models on ISIC2018.

Model	Type	Params (M)	FLOPs (G)	Dice (%)	IoU (%)	FPS
TransUNet [8]	Hybrid-Trans	105.3	31.2	90.8	83.2	18
SwinUNet [9]	Pure-Trans	27.2	5.9	92.1	85.7	35
MedFormer [37]	Hybrid-Trans	63.4	18.7	91.8	84.9	42
MedT [25]	Gated-Trans	1.6	0.4	89.3	80.9	95
UNeXt [28]	Pure-MLP	1.47	0.23	89.6	81.2	140
U-MLP [27]	Hybrid-MLP	18.9	4.2	90.4	82.6	68
**FocusGate-Net (Ours)**	**Hybrid-MLP**	**21.41**	**4.8**	**92.47**	**86.36**	**46**

### Cross-modal generalization study

To evaluate the generalization capability of FocusGate-Net across different imaging modalities and anatomical structures, we conducted experiments on additional datasets representing diverse medical imaging scenarios:

#### Dataset description.

- **BraTS 2020 (MRI):** Brain tumor segmentation dataset with 369 multi-modal MRI scans (T1, T1ce, T2, FLAIR). We focused on whole tumor segmentation [49].
- **COVID-19 CT Segmentation:** 100 CT scans with COVID-19 infection segmentation masks from the MedSeg dataset [50].
- **DRIVE (Fundus):** Retinal vessel segmentation dataset with 40 fundus images, evaluating fine structure segmentation capability [51].

#### Experimental setup.

For fair comparison, we used the same preprocessing pipeline: resizing to 256×256 and normalization. Models were trained from scratch on each dataset using identical hyperparameters. For BraTS, we used 2D slices and selected the modality with highest contrast (T1ce) (Table 5).

**Table 5:** Cross-modal generalization performance of FocusGate-Net.

Dataset	Modality	Dice (%)	IoU (%)	Precision (%)	Recall (%)
ISIC2018	Dermoscopy	92.47	86.36	93.69	92.41
PH2	Dermoscopy	97.25	94.66	98.11	96.42
Kvasir-SEG	Endoscopy	94.83	90.35	93.04	96.80
BraTS 2020	MRI (T1ce)	88.92	80.14	91.23	87.65
COVID-19	CT	85.37	74.52	88.91	82.14
DRIVE	Fundus	82.16	69.78	85.43	79.32

The results demonstrate that FocusGate-Net maintains competitive performance across diverse modalities, with expected performance variations based on task complexity. The model shows particularly strong performance on datasets with clear object boundaries (dermoscopy, endoscopy) while facing challenges in fine vessel segmentation (DRIVE) and volumetric data (BraTS, COVID-19).

### Quantitative summary of segmentation metrics

**Fig 6:**
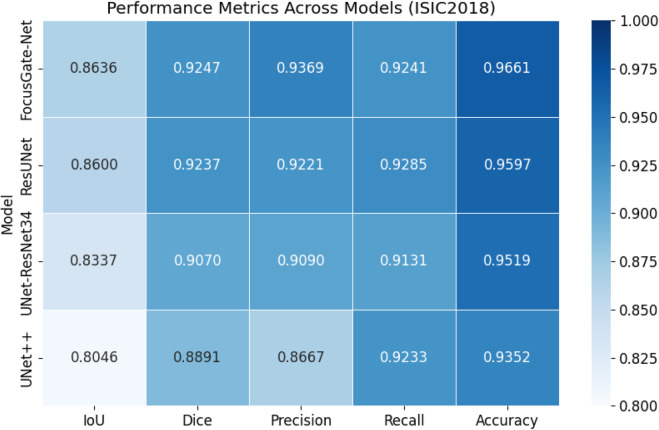
Heatmap of evaluation metrics across models on the ISIC2018 dataset. FocusGate-Net achieves the best overall performance across all metrics, particularly in Dice, Precision, and Accuracy, demonstrating its effectiveness in segmenting challenging lesion boundaries.

A comprehensive comparison of segmentation performance across different models is presented in Fig 6. The heatmap visualizes the normalized values of five key evaluation metrics: IoU, Dice, Precision, Recall, and Accuracy, based on the ISIC2018 test set.

FocusGate-Net consistently outperforms the baseline models, with the highest Dice score (0.9247), Precision (0.9369), and Accuracy (0.9661). While ResUNet performs competitively, the added attention mechanisms and hybrid design of FocusGate-Net lead to superior overall performance. These results validate the robustness and clinical applicability of our proposed architecture.

### Discussion

These results confirm that each architectural component in FocusGate-Net plays a distinct and synergistic role in enhancing segmentation performance. The shifted MLP blocks offer efficient global context modeling, enabling the network to capture long-range dependencies with minimal computational overhead. Meanwhile, the dual attention mechanisms—CBAM and Attention Gate—serve to selectively emphasize semantically meaningful regions, thus improving boundary delineation and reducing false positives.

The robustness and adaptability of FocusGate-Net were demonstrated through consistent performance across multiple challenging datasets (ISIC2018, PH2, and Kvasir-SEG), outperforming widely used architectures such as UNet, UNet++, and ResUNet. This cross-dataset generalization suggests that the model is less susceptible to domain shift, a common challenge in medical image segmentation.

From a clinical perspective, the high Dice score (92.47%) and IoU (86.36%) obtained on the ISIC2018 test set indicate that FocusGate-Net can deliver precise lesion boundary predictions, which are essential for diagnostic accuracy in dermatological assessments. Furthermore, the model’s low inference time (less than 22 ms) supports its deployment in real-time scenarios, including mobile health platforms and point-of-care systems in resource-constrained environments. These attributes collectively underscore the model’s practicality, scalability, and translational potential for real-world clinical applications.

## Conclusion

In this paper, we introduced FocusGate-Net, a novel hybrid network that combines the strengths of shifted token MLP modules, convolutional feature extractors, and dual-attention mechanisms for medical image segmentation. Our experimental results demonstrate that this architecture effectively addresses the challenges of accurate boundary delineation and feature representation in diverse medical imaging contexts. The ablation studies confirm that each component of our architecture contributes significantly to the overall performance. The shifted MLP modules capture global contextual information efficiently, while the dual-attention mechanisms (CBAM and AG) ensure focus on semantically relevant regions. Together, these elements create a synergistic effect that results in superior segmentation performance compared to conventional architectures. The cross-dataset evaluation highlights FocusGate-Net’s impressive generalization capabilities, with high performance on both dermatological (PH2) and gastrointestinal (Kvasir-SEG) datasets despite being trained primarily on ISIC2018. This transferability is particularly valuable in clinical settings where models must adapt to variations in imaging devices and protocols. When compared to state-of-the-art architectures, FocusGate-Net achieves the highest Dice score (92.47%) and IoU (86.36%) on ISIC2018, while maintaining competitive inference speed and parameter efficiency. The lightweight variant (C16-256) demonstrates that our architectural principles can be scaled down for deployment on resource-constrained devices without severely compromising performance. Future work will focus on extending FocusGate-Net to 3D medical image segmentation tasks and exploring self-supervised pre-training strategies to further improve performance with limited labeled data. Additionally, we plan to investigate the incorporation of uncertainty estimation to enhance clinical decision support capabilities. In conclusion, FocusGate-Net represents a significant advancement in medical image segmentation, offering a balanced approach that combines accuracy, efficiency, and generalizability. We believe this architecture will contribute to improved diagnostic capabilities in clinical practice and inspire further development of hybrid models that leverage the strengths of different neural network paradigms.
